# Unraveling the nature of autism: finding order amid change

**DOI:** 10.3389/fpsyg.2015.00359

**Published:** 2015-03-30

**Authors:** Annika Hellendoorn, Lex Wijnroks, Paul P. M. Leseman

**Affiliations:** Department of Special Education, Centre for Cognitive and Motor Disabilities, Utrecht University, Utrecht, Netherlands

**Keywords:** autism spectrum disorder, invariance detection, perceptual learning, embodied cognition, neural plasticity

## Abstract

In this article, we hypothesize that individuals with autism spectrum disorder (ASD) are born with a deficit in invariance detection, which is a learning process whereby people and animals come to attend the relatively stable patterns or structural regularities in the changing stimulus array. This paper synthesizes a substantial body of research which suggests that a deficit in the domain-general perceptual learning process of invariant detection in ASD can lead to a cascade of consequences in different developmental domains. We will outline how this deficit in invariant detection can cause uncertainty, unpredictability, and a lack of control for individuals with ASD and how varying degrees of impairments in this learning process can account for the heterogeneity of the ASD phenotype. We also describe how differences in neural plasticity in ASD underlie the impairments in perceptual learning. The present account offers an alternative to prior theories and contributes to the challenge of understanding the developmental trajectories that result in the variety of autistic behaviors.

“The art of progress is to preserve order amid change, and to preserve change amid order.”

(Whitehead, 1929, p. 339)

After decades of intensive research in the field of autism spectrum disorder (ASD), it is still unclear what causes this disorder and how this disorder emerges. Although several theories have been proposed to explain ASD, current approaches are not able to account for the myriad of symptoms and heterogeneity present in ASD. The three most influential cognitive theories (Rajendran and Mitchell, 2007) that have been proposed are the Theory of Mind (ToM; theory; Baron-Cohen et al., 1985), the Executive Dysfunction hypothesis (Ozonoff et al., 1991), and the Weak Central Coherence (WCC) account (Frith and Happé, 1994; Happé and Frith, 2006). The first two theories attribute the symptoms of ASD to a deficit in an internal cognitive structure or module, while the latter explains ASD by a detail-focused processing style. According to these theories, learning is a top-down cognitive process and the construction of meaning is located in the mind of an individual. Even the WCC account, which has contributed to a shift toward perceptual processes in ASD, considers perception as a top-down cognitive process (De Jaegher, 2013). Although these approaches have contributed significantly to the understanding of ASD, they are less able to account for the fact that ASD is a *pervasive developmental* disorder (Mottron et al., 2006; Rajendran and Mitchell, 2007). That is, they have difficulties to explain both the social and non-social symptoms and their explanations do not start from a developmental perspective, but are rather limited to a static impairment (Rajendran and Mitchell, 2007; López, 2013). A developmental approach that suggests a deficit in a domain-general learning process instead of a static module might extend our understanding of how the ASD phenotype emerges in the first years of life and develops over time. Our theoretical approach to ASD is grounded in Gibson’s perception-action theory (Gibson, 1979/1986; Gibson and Pick, 2000), the theoretical framework of embodied embedded cognition (Varela et al., 1991; Smith, 2005; Smith and Gasser, 2005; Barrett, 2011), and the neural mechanisms of pruning and synchronization (Uhlhaas and Singer, 2006; Hyde et al., 2010; Friston, 2011; Miller and Buschman, 2013). Our central hypothesis is that children with ASD are born with a deficit in detecting invariant structures and that differences in the ability to detect invariants might explain the heterogeneity in the ASD phenotype.

Invariance detection means selective attention to the relatively stable patterns or structural regularities in the changing stimulus array (Gibson and Pick, 2000; Gogate and Hollich, 2010). Invariance detection helps people to perceive and experience order in their surrounding world which has changing and invariant properties. An example of such an invariant is constancy of size. This means that an object’s size does not vary as the observer’s viewing distance or viewing angle changes. Actions of people also contain invariant spatiotemporal properties. These invariants differentiate a specific action from other actions. A walking movement, for instance, is specified by a specific spatial configuration of a human form in a specific pattern of change over time. Invariants can be divided in structural and transformational invariants (Michaels and Carello, 1981; Mossio and Taraborelli, 2008). Elements that remain the same over change are referred to as structural invariants. For example, the shape of an object remains the same as it rotates. Transformational invariants are structures that specify the *change* in an object or person over time. This is also referred to as change *constancy* (Michaels and Carello, 1981). The bouncing of the ball and the walking movement of a person are examples of transformational invariants. Despite enormous variation, the movements of bouncing and walking are easily recognized (Kim et al., 1995).

The invariant structure in the environment is intelligible as offering an agent a certain type of interaction (Gangopadhyay and Schilbach, 2012). In other words, the invariant structure specifies the action possibilities in the environment. These possibilities are referred to as affordances (Gibson, 1979/1986; Gibson and Pick, 2000). It is expected that difficulties in invariance detection in ASD result in being slower and less able in perceiving the affordances of the environment. In contrast to the aforementioned ASD theories, this is not considered a top-down cognitive process that locates the construction of meaning in the mind of an individual in the form of internal representations acquired by detached passive observation. Rather, detecting invariance and affordances is considered an embodied embedded learning process that can only emerge from the active interaction of an agent with its environment. Cognition cannot be separated from perception and action; it emerges from, is embedded in, and may be equated with perception-action processes (Smith and Gasser, 2005; De Jaegher and Di Paolo, 2007; Smith and Sheya, 2010).

The very basic perceptual learning process of invariance and affordance detection, which we share with animals (Hauser et al., 2001; Barrett, 2011; Wood, 2013), is already present at birth (Gibson and Pick, 2000; Teinonen et al., 2009). This process is the fundamental starting point for infants to perceive order, to understand and act upon their highly complex physical and social environment of objects, events and persons (Gibson and Pick, 2000; Fiser and Aslin, 2001, 2002a,b, 2005; Gómez, 2002; Gogate and Hollich, 2010; Gangopadhyay and Schilbach, 2012). The perception of invariants reduces uncertainty. A general purpose of most organisms is to make the environment as predictable as possible, because when one needs only to attend to information in the environment that is unexpected, one does not have to waste energy (Friston, 2011; Hohwy, 2012; Clark, 2013). This is an economical principle in which energy is only used for attending to events that are unexpected. This means that if nothing is expected, because one is not able to detect invariants, all incoming information is attended to and in turn this easily will lead to a sensory overload and feelings of being overwhelmed and exhausted (Pellicano, 2013). Detecting invariants reduces uncertainty, makes the environment more predictable and will increase feelings of security. Moreover, infants will experience that their actions can lead to predictable outcomes. As a result they will develop expectations about the predictability and controllability of their actions and experiences, hence a sense of control (Gibson and Pick, 2000).

Invariance detection at a more basic level serves as a building block for invariance detection at more complex levels (Gogate and Hollich, 2010). Through active engagement with the environment in continuous perception-action cycles children will be able to detect ever more complex affordances eventually, which is comparable to the way experts are able to perceive affordances unavailable to novices (Gibson and Pick, 2000). Central to our hypothesis is that we propose that from birth on infants with ASD experience difficulties in detecting invariants and as a result the core symptoms of ASD will gradually emerge. We propose that the core symptoms of ASD emerge after many experiences with the environment during which even simple invariants and affordances may not be detected or very slowly. The detection of lower- order invariants serves as a building block for the detection of higher-order invariants and affordances (Warren and Shaw, 1985; Best, 1995; Wells, 2002; Gogate and Hollich, 2010). Additionally, the perception of invariance at a basic level provides order, structure and a sense of control. This is a premise for further exploration, which facilitates the detection of other invariants and affordances. On the contrary, when the world is perceived as largely unpredictable and chaotic as a result of not being able to detect even simple invariants, this will hinder exploration and thereby the detection of other invariants and affordances.

As outlined above, a deficit or delay in invariance and affordance detection at birth in ASD, may lead to a cascade of consequences. Our hypothesis suggests that it will lead to various degrees of impairments in every developmental domain, from sensorimotor development to language and social development. This hypothesis may explain the wide variety of ASD impairments in different developmental domains instead of being limited to only on a subset of behaviors. Moreover, we suggest that the severity of autistic symptoms is determined by the degree of impairment of this particular learning process. That is, the less well the process is functioning, the more severe the symptoms will be and vice versa.

In the present paper, we will first discuss a range of empirical studies pertaining to several developmental domains that are in accordance with our hypothesis. Then, some specific neural mechanisms that are in line with our hypothesis are discussed. Thirdly, we will explain how developmental cascades may play a role in the development of ASD. We will also address the spectrum nature of autism. Finally, research designs for future research and the possible clinical implications of our hypothesis will be discussed.

## Motor Functioning and Imitation

Although not considered a core symptom of ASD many children and adults with ASD display delays and impairments in motor functioning, including delays in motor milestones, atypicalities in motor coordination, postural control, anticipation, planning and symmetry of movements (Fournier et al., 2010; Staples and Reid, 2010; Bhat et al., 2011; Forti et al., 2011; Brisson et al., 2012). We hypothesize that part of these motor problems may be explained by difficulties in the perception of invariants. Actions can be specified by an invariant structure combined with variant features. The invariant structure of a movement remains essentially the same while other features are changing, for instance when adjusting to a changing environment (Schmidt and Wrisberg, 2008). Research shows that invariant patterns are inherent in our everyday actions (Baldwin et al., 2008), such as toothbrushing (Reed et al., 1995) and locomotion (Das and McCollum, 1988; Funato et al., 2010). Reed et al. (1995) explain: “What remains invariant in performing a task from time to time and place to place is not the sequence of units, nor even all the particular units, but rather a higher order relationship among units.” (p. 44). Invariants characterize the total movement. A reaching movement, for instance, is characterized by an arm moving away from the body, approaching the object closer and closer, and closing in as it begins to make contact (Gibson and Pick, 2000, p. 134). The process of learning a specific motor skill is associated with a reduction in trajectory irregularity (Shmuelof et al., 2012). Being attuned to the invariant structure is necessary to predict and anticipate an action (Gibson and Pick, 2000).

Research shows that children with ASD do not make anticipatory postural adjustments in a bimanual lifting task and in a feeding situation, which indicates the use of a feedback rather than a feedforward mode of control (Schmitz et al., 2003; Brisson et al., 2012). The use of a feedforward mode of control requires detecting the invariant structure of the action. A deficit in the detection of the invariant structure of the total movement may also explain why children with ASD have difficulties in chaining motor acts into a global action (Cattaneo et al., 2007; Gordon and Stark, 2007; Gidley Larson and Mostofsky, 2008; Fabbri-Destro et al., 2009). Children with ASD also often imitate a movement only partially and need more attempts to imitate the whole action (Vanvuchelen et al., 2007, for a review, on imitation in autism see Vanvuchelen et al., 2013). These findings are also consistent with research that indicates that children with ASD require additional demonstration and practice to learn how to retrieve a gift from a box, a task that requires a specific movement trajectory (Nadel et al., 2011). This latter study indicates that people with ASD are to some extent able to detect the invariant structure of an action, but are attuned more slowly to this structure. Other studies support that increased exposure to an action sequence is necessary to learn an action sequence to individuals with autism (Gordon and Stark, 2007). This indicates that at least some individuals with ASD are able to detect the invariant structure of an action, but are much slower in doing this than individuals without ASD. Consistent with the invariance detection hypothesis, children with ASD are performing better when imitating the actions of a robot compared to human movement (Pierno et al., 2008). It is clear that robotic movements contain less variance compared to human actions, which are characterized by a high degree of variability around the invariant structure (Pierno et al., 2008). Assuming that the actions of typically developing people are guided by the ability to pick up the invariant structure, while invariants are not or more slowly detected by people with ASD, more and longer lasting variability would be expected in the ASD group and less movement variability in the typically developing people. Studies indeed indicate that people with ASD display greater movement variability (larger standard deviations) in a precision grip task, in a reach-to-grasp task and in gait compared to the control group (Mari et al., 2003; Rinehart et al., 2006; David et al., 2009).

## Perception and Sensory Sensitivities

It has been reported that people with ASD display superior change detection (Smith and Milne, 2008), reduced change blindness (Fletcher-Watson et al., 2012), and are more successful at visual search tasks (Kaldy et al., 2011) compared to typically developing controls. Smith and Milne (2008) found that people with ASD were superior in the detection of change in a film clip and this advantage was particularly apparent for the “marginal” changes; changes to items not highly relevant to the scene. At first site this finding contradicts our hypothesis, but this sensitivity to change might be explained by the difficulties people with ASD have with detecting the invariant structure of the scene. In typically developing individuals detection of the invariant structure of the scene helps to ignore the perception of changes in irrelevant items. The result of invariance detection process is the separation between the relevant items building the invariant structure and the irrelevant items that are not part of the invariant structure. This may also explain the faster performance of individuals with ASD on tasks such as the block design task scales (Shah and Frith, 1993) and faster and more accurate performance on the embedded figures task (EFT; Jolliffe and Baron-Cohen, 1997) compared to matched controls. Furthermore, individuals with autism discriminate better between novel, highly similar stimuli in a perceptual learning task than do a matched control group (Plaisted et al., 1998). All three tasks require detection of a target between distractors and are determined by the degree of similarity between target and distracters. Typically developing individuals have more difficulties detecting the target, since the target is not part of the invariant structure or pattern among the distractors or of the larger figures in the EFT. The target is not expected and attended to. Typically, the detection of the invariant structure also leads to change blindness. In this way, the discovery of invariant structure yields economy in perception. It serves as a selection mechanism (Gibson and Pick, 2000). Interestingly, Fletcher-Watson et al. (2012) did find reduced change blindness in children with ASD in complex social scenes. Other studies, however, did not find evidence of attenuated change blindness using pairs of images of isolated objects in a similar age sample (Burack et al., 2009). These contrasting findings have been explained by the difference in using social versus non-social information (Fletcher-Watson et al., 2012). However, this may also be explained by *the nature of* social information. In a complex social scene people with ASD may experience large difficulties in detecting the invariant structure as such a scene has many features and extracting regularities and therefore is highly complex. In contrast, extracting the invariant structure of isolated objects is far less demanding and most children with ASD are apparently able to do that. Although children with ASD do show change blindness with isolated object stimuli they do not with more complex stimuli. This can be explained by a general deficit in the detection of invariant structure. The idea that impaired invariant detection plays a role in the perceptual abilities of people with ASD is also consistent with findings regarding deficits in motion detection in ASD (Annaz et al., 2010). In order to detect motion one needs to be able to detect the transformational invariant. Studies show that children with ASD are less able to perceive biological motion than typically developing children when asked to identify a human point-light walker from scrambled dots (Blake et al., 2003). Identifying biological motion requires detecting both structural invariants (e.g., human body shape) as well as transformational invariants (e.g., pattern of change that specifies human walking).

Children with ASD have been found to orient more to non-social stimuli than to social-biological motion (Klin et al., 2003). A possible explanation is that non-social contingencies often require extracting regularities from fewer features, over shorter temporal and spatial scales. Annaz et al. (2010) found that in contrast to typically developing children who demonstrated increased sensitivity to biological motion with increasing age, no evidence of learning took place in children with ASD over time, demonstrating more difficulties detecting the invariant structure of biological motion (Annaz et al., 2010).

If the detection of invariant structure is impaired in ASD, one would also expect to find impairment in categorization and prototype formation because categorization and prototype learning requires extraction of the invariant common structure of several instances of a category. Several studies indeed show deficits in prototype formation and categorization, both with social and non-social stimuli (Klinger and Dawson, 2001; Klinger et al., 2007; Gastgeb et al., 2009, 2012; Church et al., 2010; Fields, 2012). Supporting our hypothesis of invariance detection, stimuli with complex dimensions of similarity are expected to be more problematic for individuals with ASD than stimuli made up of simple binary feature combinations (Bott et al., 2005; Molesworth et al., 2005, 2008). Many social-communicative stimuli, including facial stimuli, gestures and prosody in language use, have these more complex and dynamic invariant structures and therefore will be difficult to detect for people with ASD. These expectations are consistent with research that shows slowed habituation to faces in toddlers with symptoms of ASD (Webb et al., 2010).

Several studies demonstrate sensory abnormalities in all sensory modalities in children with ASD which range from hypersensitivity to hyposensitivity (Pellicano, 2013; for a meta-analysis, see Ben-Sasson et al., 2009). Our hypothesis of a deficit in invariance detection may explain several of these abnormalities. Since typically developing people are able to detect the regularities in the environment and therefore able to predict and anticipate, information attended to will be reduced and sensory information will be predicted and anticipated. Because people with ASD may have, as we suggest, difficulties in detecting the invariant structure, they are less able to predict and anticipate and therefore will be more often “surprised” by for instance a noise or a touch compared to typically developing people. Not being able to detect invariant structures will lead to less selection and compression of information, and reduction of irrelevant information and consequently to regular experiences of sensory overload and strong feelings of uncontrollability (Ben-Sasson et al., 2009; Pellicano, 2013). This is also consistent with findings that people with ASD do not seem to show these sensitivities in self-produced or self-controlled sensory effects (Pellicano, 2013).

## Repetitive and Stereotyped Behavior

Repetitive and stereotyped patterns of behavior, interests and activities are a core feature of ASD (Watt et al., 2008). As already stated before, if people with ASD are not attuned to the regularities in the environment, their environment becomes rather unpredictable and they will not achieve a sense of control. They rather develop an aversion to variance and try to avoid change. Repetitive and stereotyped behavior may therefore be adaptive for people with ASD. A preference for simple invariant structures of regularities, either self-produced or perceived, might be viewed as a strategy to seek environments and events which are highly predictable. As a well-known ASD autobiographical writer with ASD describes: “The constant change of most things never gave me any chance to prepare myself for them. Because of this I found pleasure in doing the same things over and over.” (Williams, 1992, p. 39–40). Repetitive manipulation of objects may provide children with ASD with feelings of security and a sense of control of an unpredictable and highly variant environment, hence reduce feelings of anxiety or anger (Leekam et al., 2011; Rodgers et al., 2012). Repetitive behaviors can be seen as performatory actions that have expected results. They depend on and confirm an already learned affordance (Gibson and Pick, 2000). Exploratory actions, in contrast, provide new and a priori variant information. The fact that children with ASD display less and less varied exploratory behavior compared to children with other developmental delays and typically developing children and (Pierce and Courchesne, 2001; Ozonoff et al., 2008; Koterba et al., 2014; Hellendoorn et al., 2015) may well be explained by their difficulties in detecting invariant structure in new information. Adding new information through exploration only creates uncertainty and is therefore avoided. Moreover, children with autism may also be unable to demonstrate complex exploratory behaviors, since the discovery of certain invariants and affordances is required to provide the gateway for the discovery of other, possibly higher-order, invariants and affordances (Gogate and Hollich, 2010). A child may for instance first discover the characteristics of two separate objects and over time discover that these objects can be combined (Lockman, 2000). If children with autism are impaired in invariant detection, they may not proceed to the level of making combinations or proceed to making combinations much later than typically developing children. An early study indeed did find that combinatorial use of toys indeed differentiates children with autism from children with an intellectual disability and typically developing children (Tilton and Ottinger, 1964). Preference for observing spinning or rotating movements (spinning objects, watching washing machine rotating, spinning wheels of toy cars), for instance, is common in children with ASD (Bracha et al., 1995). A rotating movement is characterized by invariance and predictability of visuotemporal-spatial information. This keeps uncertainty to a minimum and provides people with ASD with a sense of control. The fact that people with ASD show intact or even strong systemizing (Baron-Cohen, 2009), is consistent with our hypothesis. Systems are characterized by clear regularities, and strict “if p, that q rules.” The regularities in our daily environment are very different from the regularities that make up systems. System regularities are lawful and deterministic, while social situations are for instance characterized by more subtle complex regularities that have to be extracted over larger temporal and spatial scales with many features.

## Language and Communication

Invariance detection is a fundamental mechanism in acquiring language and communication (Saffran and Wilson, 2003; Gogate and Hollich, 2010; Romberg and Saffran, 2010). Although young infants do not yet understand the language addressed to them, language already develops by the detection of the phonotactic invariants. Young infants (even before they are born) already detect the acoustically invariant patterns (for instance the recurrent rhythm and melody) that specify their native language (Kuhl, 2000; Karmiloff and Karmiloff-Smith, 2001). An important mechanism, crucial for language acquisition, is the ability to identify phonemes and phoneme clusters despite variation in speakers, background noise, speech rate, and surrounding phonemes. Based on detecting stress patterns and the statistical distribution patterns of phoneme clusters, infants become able to segment the speech stream in units corresponding with words and phrases in the first years of life, which underlies word learning (Gómez, 2002; Saffran and Wilson, 2003; Romberg and Saffran, 2010). On the basis on the relative invariance in the syllabic constituents and the syllable stress patterns of their names, infants recognize their own names earlier than those of others (Mandel et al., 1995; Gogate and Hollich, 2010). Invariance detection also underlies grammar learning, which according to the construction grammar approach, is based in abstracting types, or categories, from varying tokens (Jackendoff, 2002; Tomasello, 2006). Children with ASD are indeed impaired in extracting phonetic features (Lepisto et al., 2008), which points to a general deficit in invariant detection. Another study supports this hypothesis demonstrating that children with ASD are impaired in the ability to detect transitional probabilities in language (Scott-Van Zeeland et al., 2010). It may also be possible that some children with ASD are able to extract the invariant structure in language, but are slower in detecting this structure and therefore need more time and exposure to linguistic information. This is consistent with the study of Scott-Van Zeeland et al. (2010), who found a deficit in implicit language learning in children with ASD listening to a 2.4 min speech stream while another study did not find a deficit in statistical language learning in children with ASD using a 21 min speech stream (Mayo and Eigsti, 2012). It should also be noted that both studies used high-functioning children with ASD. It can be expected that lower functioning children with ASD would also be impaired in extracting the invariant structure in the lengthy speech stream presented in the second study. Since invariance detection is already present in the first months of life (Kuhl, 2000; Teinonen et al., 2009) it can be expected that children with ASD show delays and deficits in language acquisition at an early age. Children with ASD indeed show significant delays and deficits in language development early in life. Those children with ASD who eventually acquire spoken language speak their first words when they are 38 months old on average (Howlin, 2003), as compared to 12–18 months in typically developing children. No babbling by 12 months and no functional words by 16 months are both used as so called red flags in early screening for ASD (Dereu et al., 2010). It is also often reported that children with ASD seem deaf, because they do not or only poorly respond to voices and their own names (Osterling and Dawson, 1994; Werner et al., 2000). Additionally, deficits in syntactic and morphological skills are present in ASD (Eigsti et al., 2007). Prosodic impairments have also been found in ASD, characterized by inappropriate use of stress, pitch variation, and rhythm (Shriberg et al., 2001; Paul et al., 2005). Studies demonstrate that some language domains are more impaired than others. Learning the meaning of abstract words classes is more impaired in ASD than the learning of concrete word classes. This has been demonstrated by behavioral studies and neuroscientific studies (Toichi and Kamio, 2003; Harris et al., 2006) and can also be attributed to a deficit in invariance detection. Learning the meaning and correct use of abstract words and use it correctly requires extracting the invariant over a large amount of uses of these words in variant contexts, while learning the meaning of single words referring to concrete objects requires less complex invariance detection.

Obviously language learning takes place in context, with the infant and caregiver surrounded by objects they can see and touch while engaging in social interactions. Romberg and Saffran (2010) conclude, for example, that language learning is most efficient when regularities in the speech stream coincide with environmental regularities. If infants with ASD indeed have a general deficit in invariance detection, it is expected that language acquisition in ASD is not only negatively influenced by difficulties in detecting invariants in speech, but also by deficits in invariance detection in the broader context of objects, events and social interaction which are also relevant for language acquisition (Yu and Ballard, 2007). Note that most of the reported language problems may also be explained by social-communicative impairments, which may also dependent on invariant detection as will be described below.

## Social Interaction

Gibson (1979/1986) stated that the richest and most elaborate affordances for people are provided by other people (p. 135). Detecting the regularities or invariants in social behavior is more difficult than detecting regularities in the physical environment (e.g., objects), because human behavior is dynamic, variable, context-dependent and not lawful and deterministic. This may explain why people with ASD experience most difficulties in the social-communicative domain, for instance with face processing. As the eye region and the muscles in the face, used to express emotions, are the most variant part of the faces, they will make the detection of invariances more difficult than recognizing objects or even moving objects and animals. This might explain why individuals with ASD spend less time looking at faces in general (Pelphrey et al., 2002; Clifford et al., 2007) and actively avoid the eye-region (Klin et al., 2003; Kliemann et al., 2012). Our hypothesis may also explains why people with ASD prefer objects over people (Klin et al., 2003) and are better in imitating robots than humans, (Pierno et al., 2008) because robots move in a much more lawful and predictable way than humans. The variability in human motion not only makes it harder for people with ASD to detect the invariants, but will lead to a preference for interacting with objects and robots instead of humans. Consequently, children with ASD will have more difficulties to correctly identify and therefore understand human actions. Indeed, children with ASD are impaired in the ability to recognize human gestures and actions and to respond appropriately to them (Dowell et al., 2009; Swettenham et al., 2013). Moreover, they are less sensitive compared to typically developing controls in discriminating between point-light biological motion and scrambled motion when briefly presented (Blake et al., 2003) and less effective in discriminating between biological motion and mechanical motion (Cook et al., 2009). This account of the social deficits in ASD is different from the traditional ToM explanation (Baron-Cohen et al., 1985) in the sense that the ToM paradigm attributes the social difficulties to a deficit in social cognition per se, while our hypothesis states that people with ASD have problems in the social-communicative domain because of the nature of social information, i.e., the complexity of variant–invariant configuration (Hellendoorn, 2014). Thus, non-social information that is equally (in) variant is expected to lead to the same difficulties.

## Neurobiological Mechanisms

Researchers have attempted to identify to neural correlates of invariance detection in typical development. However, implicit perceptual learning does not produce a single characteristic signature of evoked neural activity in one brain system in a single direction (Reber, 2013). Studies demonstrate that changes in the brain that follow perceptual learning are spread across the brain and in different directions (for a review, see Reber, 2013). It has been demonstrated that implicit perceptual learning is not supported by the same specific structures that underlie explicit learning, such as the medial temporal lobe system (Verfaellie et al., 2013). Several studies indicate that implicit perceptual learning is based on general neural plasticity mechanisms, i.e., processes that modulate changes in connections over time (for a review, see Reber, 2013). An important neural plasticity mechanism during perceptual learning is neural synchrony (Gotts et al., 2012). Greater neural synchrony is for instance seen when people are repeatedly exposed to a stimulus (Ghuman et al., 2008; Gotts et al., 2012). Repeated exposure to a stimulus facilities its processing. With repeated exposure people become faster and more accurate in identifying the stimulus and classifying it. This perceptual learning process is a result of enhanced communication between distinct cortical regions. Repeated object classification leads to decreased neural responses in the prefrontal cortex and temporal cortex. This decrease in absolute activity is accompanied by greater neural synchrony between these regions with repetition (Ghuman et al., 2008). Several researchers have suggested that this neural synchrony in the brain reflects structures in the stimulus environment (Friston, 2011; Brette, 2012). In a heterogeneous neural population, synchrony patterns represent structure, that is, sensory invariants in stimuli (Friston, 2011; Brette, 2012). While individual neural responses vary with many aspects of stimuli, the spatial structure of synchrony is invariant (Brette, 2012). This is consistent with the idea that the brain does not construct information from the input, but rather resonates to the structured information present in the environment (Gibson, 1979/1986). Rodriguez et al. (1999) found a pattern of synchrony between occipital, parietal and frontal areas during face recognition in human, while it was absent when faces were not recognized. Since face recognition is based on the perception of a global, invariant structure (Goffaux and Rossion, 2006), the study by Rodriguez et al. (1999) provides evidence for the idea that neural synchrony patterns reflect invariant structure. Reduced neural synchronization has also been associated with deficits in gestalt perception (Uhlhaas et al., 2006). Other studies also confirm that following experience/training, firing patterns in primary visual cortex and in primary auditory cortex neurons change so that they reflect the statistical, invariant structure of the frequently experienced input patterns in a specific task (Li et al., 2004; Polley et al., 2006). If neural synchrony is a mechanism for invariance detection then dysfunctional neural synchrony in ASD would be consistent with the hypothesis that individuals with ASD have a deficit in invariance detection. Indeed, several studies support the hypothesis of dysfunctional neural synchronization in ASD (Uhlhaas and Singer, 2006, 2007, 2012; Wilson et al., 2007; Sun et al., 2012). One study, for example, demonstrated impaired prefrontal gamma band synchrony in ASD during gaze cueing (Richard et al., 2013). Interestingly, abnormal neural synchrony has also been observed in other disorders that are comorbid to ASD, such as schizophrenia and epilepsy (Uhlhaas et al., 2006; Uhlhaas and Singer, 2006, 2012). Synchronized neural oscillations are a fundamental mechanism for the emergence of coordinated networks in the brain. If these oscillatory signals are synchronized, they form ensembles and these ensembles form larger functional networks (Miller and Buschman, 2013). Neural synchrony also plays an important role in the strengthening and pruning of connections (Uhlhaas and Singer, 2011). The mechanism of dysfunctional neural synchrony is in line with the brain connectivity theories that suggest that in ASD long-range connections between brain areas are underdeveloped, while short-range connections within brain areas are overdeveloped (Just et al., 2004; Courchesne and Pierce, 2005a,b; for a review, see Wass, 2011). If the combination of too many short range connections with too little long-range connection is related to the proposed deficit in invariance detection, then it can be expected that the balance between short and long range connections is related to ASD severity. It has indeed been demonstrated that with increasing ASD severity, short-range coherence is more pronounced and long-range coherence more decreased (Barttfeld et al., 2011).

Several studies demonstrated differences between individuals with and without ASD in neural plasticity during perceptual learning tasks (Schipul et al., 2012; Dovgopoly and Mercado, 2013; Church et al., 2015). In a task where adults learned to categorize dot patterns, adults with ASD showed less changes in cortical activation over time compared to typically developing adults (Schipul, 2012). A positive relationship was found between the disruption of learning-induced changes in cortical activation and the severity of ASD symptoms. Thus, disrupted cortical reorganization seems to be related to impairments in—or slower perceptual learning in individuals with ASD. Another example of a neural plasticity mechanism that has been specifically associated with learning-induced changes in cortical activation is repression suppression (Ewbank et al., 2014). Typically, repetitions of the same stimulus result in a reduction in the neural response. This process is called repression suppression. Studies show that individual differences in autistic traits predict repression surpression in the visual cortex in perceptual learning tasks including both social and non-social visual objects and including both complex and simple shapes (Ewbank et al., 2014). The more autistic traits, the less repression suppression. In normal development, another process that is part of neural plasticity as a result of perceptual learning, is pruning. Research suggests that atypical synaptic pruning might lead to deficits in invariance detection. Studies demonstrate that when connectivity is too high, it can lead to an impaired ability to generalize and to efficiently select relevant information (Belmonte and Yurgelun-Todd, 2003; Cohen, 2007). Thus, a deficit or delay in pruning may be a potential mechanism in the proposed deficit of invariant detection in ASD. Research demonstrates an excess in synapses in children with ASD, which is consistent with a deficit or delay in synaptic pruning (Hardan et al., 2006; Hyde et al., 2010). Cortical thickness has been hypothesized to reflect pruning. Especially at a younger age children with ASD have increased cortical thickness compared to typically developing children (Hardan et al., 2006). Increased cortical thickness in ASD has been demonstrated in the primary visual and auditory cortex (Hyde et al., 2010; Nebel et al., 2014). Consistent with these results, several other studies have found increased head circumference and brain enlargement in ASD, which is also especially apparent in the first years of life (Courchesne et al., 2003; Redcay and Courchesne, 2005; Vaccarino and Smith, 2009; Schumann et al., 2010). In particular early maturing brain areas, such as the sensory and motor brain areas are under stronger genetic influence. Several researchers have suggested that differences in pruning in ASD may be partly caused by genetic differences, such as microdeletion syndromes (Fahim et al., 2012; Persico and Napolioni, 2013). An increased head circumference has also been reported for infant siblings of children with ASD (Constantino et al., 2010).

Recently, using Bayesian models, several authors have suggested that the brain implicitly generates hypotheses based on prior knowledge (Friston, 2011; Brown and Brüne, 2012; Clark, 2013). These priors are based on the detected invariant structure in the environment (Brown and Brüne, 2012; Pellicano, 2013). If priors are based on invariants in the environment and people with ASD have a deficit in invariance detection, then it can be expected that they fail to generate expectations or have priors that are not accurate (Pellicano and Burr, 2012). As a result it can be expected that people with ASD are more often than other people in a state of surprise, experiencing the world as unpredictable (Gomot and Wicker, 2012). This also may explain why people with ASD engage in behavior that minimizes change and seek for input that is highly predictable. It is consistent with the reported feelings of sensory overload in people with ASD, because the implicit model also acts as a filtering mechanism (Pellicano, 2013).

In contrast to cognitivist neuroscience, current embodied cognition approaches consider internal states (resonating invariant structure in the environment) not as representations, but rather as “action-oriented pointers” or “sensorimotor activity patterns” (Engel et al., 2001; Clark, 2013). This is consistent with the aforementioned idea that invariant structures in the environment specify affordances. Thus, people with ASD do not perceive the same affordances as other people because of their deficit in invariant detection. Recent neuroscientific findings suggest that the brain is making *action-oriented* predictions specified by invariant structures (Friston, 2011; Clark, 2013). Research with monkeys, for example, shows that the brain directly codes the external world in terms of action possibilities by canonical neurons that fire when the monkey observes an object and mirror neurons that fire when the monkey observes another monkey or human being perform a goal directed action (Gallese, 2007; for a review, see Rizolatti and Craighero, 2004). The canonical and mirror neuron system may be an example of a system that consists of synchronized oscillations reflecting the invariant structure of objects and actions of people and then specifies affordances directly. Although evidence on mirror neurons in humans is still scarce (for a review, see Hamilton, 2013), some studies with brain imaging techniques, indicate the existence of both canonical and mirror neuron systems in humans (Keysers and Gazzola, 2009; Kilner et al., 2009). Some studies have suggested that children with ASD have an impairment in the mirror neuron mechanism (Dapretto et al., 2006), which is also known as the “broken mirror” hypothesis (Oberman et al., 2008). Other studies suggest that that mirror neuron system may not be broken down but just more slowly developing in ASD (Bastiaansen et al., 2011). The functioning of the mirror neuron system is dependent on the communication between brain areas (Kilner et al., 2009), therefore the proposed mechanism of impaired neural synchrony in ASD may also impair the mirror neuron network.

## Developmental Cascades Across Domains

In studying ASD and other developmental disorders, it is important not to rely on phenotypic outcomes to draw conclusions about impaired or intact modules in the initial state, but rather to take a developmental approach and examine the course of the disorder over time, including the role of developmental cascades across domains (Paterson et al., 1999; Masten and Cicchetti, 2010). The present paper suggests that a deficit in invariant detection in ASD is already present at birth and it will have a great impact on the child’s development because invariant detection develops from detecting few and lower order invariants to the ability to detect more and more complex higher order invariants and affordances. Not only is invariance detection a core mechanism of learning and development that affects all developmental domains, it is also expected that development in one domain will influence the development in other domains (Paterson et al., 1999; Masten and Cicchetti, 2010). This is consistent with an embodied cognition perspective and a dynamic systems approach to development, which considers behavior as the emergent pattern of multiple interacting and cooperating components (Thelen, 2008). Several examples of plausible developmental cascades have been described in typically developing children, such as relationships between motor functioning, exploration, spatial cognition, perception, social interaction and language development (Campos et al., 2000; Clearfield et al., 2008; Iverson, 2010; Soska et al., 2010; Clearfield, 2011; Oudgenoeg-Paz et al., 2012; Smith, 2013; Wellsby and Pexman, 2014). These developmental cascades should also be considered when studying the development of ASD symptomatology. Indeed, several studies have suggested and demonstrated interrelationships between developmental domains in ASD, for instance between motor imitation and language development (Stone and Yoder, 2001), exploration and language (Hellendoorn et al., 2015), motor skills and social interaction (Bhat et al., 2011), visual processing and social skills (Hellendoorn et al., 2014), and joint attention and language development (Murray et al., 2008).

## Severity of Autism

Autism is a *spectrum* disorder which means that the severity and nature of symptoms vary greatly among people with autism. Our hypothesis of a deficit in invariance detection would predict a continuum and not the development of a discrete disorder. We assume a direct link between the ability to detect invariants and the severity of the symptoms. We further assume that the ability to detect invariant structures will be determined by synaptic pruning and processes of neural synchrony. Moreover, we expect that the ability to detect invariance is intact in all people with ASD, at least at a minimal level of functioning, as we share as human beings this fundamental learning process with many other animals. Finally, only a fully intact ability to detect invariances contributes to a normal development. From a dynamic system perspective and embedded cognition approach, stressing the coupling between heterogeneous systems, we assume that even a slightest deviation in this process may lead to atypical developmental trajectories in many domains. While some children with ASD, who function at a very low level of development, may just be able to detect invariance over little variability, and therefore engage mainly with static objects, demonstrate repetitive and stereotyped behaviors and are highly reliant on a predictable and structured environment in order to function relatively well, other children with ASD may be able to cope with variability much better, which makes them able to interact socially. This latter group will be more flexible in adapting to changing situations and contexts. The severity of ASD symptoms is also dependent on the presence of a comorbid intellectual disability. While implicit learning performance, such as invariance detection, has been shown to be unrelated to intelligence quotient (IQ), explicit learning is strongly (positively) correlated with IQ (Gebauer and Mackintosh, 2007; Kaufman et al., 2010). Therefore, people with ASD with normal or high intelligence may compensate for their implicit learning deficit by using explicit learning processes. Studies demonstrate that when explicit social cognition task are used, ASD and typically developing (TD) individuals do not differ in social cognition, while they did differ in implicit social cognition (Callenmark et al., 2014). In addition, some studies indicate that persons with ASD are able to achieve equivalent performance on tasks that are focused at measuring implicit learning, because they are able to solve the task by using explicit strategies (Ozonoff and Miller, 1995; Klinger and Dawson, 2001). This may explain part of the heterogeneity and varying levels of functioning in ASD. People with ASD with normal intelligence levels may be able to explicitly learn some of the regularities that they do not perceive automatically, Moreover, they can be instructed how to act accordingly, but they are not able to learn the more subtle implicit social rules. Although this strategy is inflexible and less efficient, it may facilitate the interaction with the environment. Individuals with ASD and intellectual disability are expected to be impaired in both implicit and explicit learning.

## Relationship with other Theories

The theory presented in this paper has both similarities to and differences with other accounts on autism. Our theory differs from the ToM theory, the executive dysfunction (EF) account and WCC account of ASD in the sense that our theory proposes a process that locates the construction of meaning in the person-environment interaction, while the other theories are top-down oriented cognitivist theories that locate the construction of meaning internally in the mind of an individual. Our hypothesis of invariant detection has similarities and builds upon other theories, such as the enhanced perceptual functioning hypothesis of Mottron et al. (2006). The latter account suggests that locally oriented perception is enhanced in ASD and that this may explain both social and non-social information processing. Our proposal is also similar to the account of Gepner and Féron (2009). They suggest that an impairment in temporo-spatial processing can explain both social and non-social characteristics of ASD. While our theory has similarities to these accounts of ASD, the theory of invariance detection moves beyond a description of a general deficit by suggesting a specific learning mechanism. Our hypothesis is also related to the theory proposed by Pellicano and Burr (2012) who suggest that attenuated Bayesian priors may be responsible for the unique perceptual experience of autistic people. This means that the perception is less modulated by prior experience in ASD. While our theory is related to this hypothesis, Pellicano and Burr (2012) focus on weak priors as the central mechanism, while we propose that invariance detection is the process that is altered in ASD. In contrast to Pellicano and Burr (2012), we do not suggest that people with ASD have weak priors, but *different* priors. Because of their invariance detection impairments, we hypothesize that the priors of people with ASD also include variant aspects of the environment and are more exemplar-based instead of prototype-based (Dovgopoly and Mercado, 2013; Church et al., 2015). Temple Grandin said in an interview in 2010: “My brain is like Google Images. If someone says the word factory, most people think of a vague place. I think in detail of every factory I ever saw^1^.” We suggest that the priors of people with ASD are less based on the stable regularities in the environment and are therefore also less useful in predicting the environment. While Pellicano and Burr (2012) mainly focus on describing the relevance of their theory in relation to sensory and perceptual features of ASD (Pellicano, 2013), we have attempted to explain the relevance of our hypothesis for several developmental domains.

## “Fractionable Triad” vs. Invariance Detection Deficit?

Our hypothesis of invariance detection, a universal deficit that may explain the different characteristics of ASD, seems to be hard to reconcile with the proposal of the “fractionable” autism triad in which it was claimed that, ASD is not a coherent syndrome (Happé and Ronald, 2008). Instead, it is hypothesized that the social-communicative impairments and the restricted and repetitive behavior and interests can be separated and have distinct causes (Happé and Ronald, 2008). This hypothesis is supported by studies that demonstrate that a two-factor solution, not a one factor solution adequately fits the data (Snow et al., 2009; Frazier et al., 2012, 2014) and by studies that suggest that the inherited influences on the domains of ASD are independent of one another (Ronald et al., 2006a,b). However, although some studies find a two-factors solution, Frazier et al. (2014) demonstrate that the two factors are strongly related. Strong intercorrelations between traits would be expected from a universal deficit model, while it could also be the case that these relations are the result of developmental cascades (Brundson and Happé, 2014). The findings that the two core ASD features are separable should not immediately be taken as evidence that the features cannot result from the same underlying cause. It could be the case that due to the use of compensatory processes, such as the use of explicit strategies, that may influence one domain more than the other, social-communicative impairments and restricted repetitive behaviors become fractionated, despite the fact that they are both caused by a deficit in invariance detection (Brundson and Happé, 2014; Williams and Bowler, 2014). Moreover, other factors, such as environment, intervention and age, also influence the severity of symptoms in every domain (Frazier et al., 2012; Brundson and Happé, 2014) and if these factors differentially affect the different domains, they may also lead to results that suggest a fractionation of these symptoms, despite having the same underlying mechanism. In addition, it is important to take into account methodological considerations. It may be the case that the social-communicative impairments and repetitive restricted behaviors are related, while no relationships are demonstrated in studies, because the tasks used to investigate the two traits do not represent the symptoms well, for example when a ToM task is not related to social skills in daily life (Brundson and Happé, 2014). All in all, while some studies find that the two traits of ASD can be separated, this does not rule out the possibility that ASD represents a coherent syndrome with a underlying deficit that is related to different symptoms, such as an invariance detection deficit.

## Invariance Detection in other Developmental Disorders

An important question is whether a deficit in invariance detection is specific for ASD, i.e., how common a deficit in invariance detection is among other developmental disorders and how it relates to intellectual disability. As mentioned before, implicit learning performance has been shown to be relatively independent of IQ level, while explicit learning is strongly correlated with IQ (Gebauer and Mackintosh, 2007; Kaufman et al., 2010). Studies demonstrate that implicit learning performance is typical in children and adults with intellectual disability, while they do more poorly on explicit learning tasks (Atwell et al., 2003; Vinter and Detable, 2008). Therefore, we hypothesize that the impairments in invariance detection in individuals with ASD are not the result of a general intellectual disability. While there are similarities between ASD and attention-deficit/hyperactivity disorder (ADHD), and between ASD and specific language impairment (SLI) it is unclear whether these disorders represent a common etiology. The question is whether a deficit in invariance detection may also underlie impairments present in ADHD or SLI or whether it is specific to ASD. Some studies suggest that a domain-general process of implicit learning is impaired in SLI, which can present itself also in non-linguistic domains (Ullman and Pierpont, 2005; Evans et al., 2009; Hsu and Bishop, 2011; Lukács and Kemény, 2014; Lum et al., 2014). This idea is supported by research that indicates that SLI is also associated with impairments in several non-linguistic functions, such as motor coordination (Hill, 2001; Zelaznik and Goffman, 2010), visuospatial processing (Akshoomoff et al., 2006; Vugs et al., 2013), and with social impairments that are also typical of ASD (Leyfer et al., 2008). These findings suggest that it is possible that impairments in ASD and SLI are both related to a deficit in invariant detection and that ASD and SLI are on a continuum. However, we need to be cautious in drawing this conclusion, since the evidence is very speculative and because other studies suggest distinct underlying mechanisms for similar impairments in ASD and SLI (Whitehouse et al., 2008; Williams et al., 2013). Individuals with ADHD also show partly similar impairments to the impairments present in ASD. ADHD is for instance associated with impairments in social cognition such as emotional face perception (for a review, see Uekermann et al., 2010) and motor coordination difficulties (Fliers et al., 2008). While some studies indicate that individuals with ADHD display impairments in implicit learning (Huang-Pollock et al., 2014), which could be an explanation for the deficits in several developmental domains, most theoretical models and empirical studies suggest that implicit learning is not the most probable underlying mechanism of the impairments in ADHD. Rather, the impairments in attention and inhibition present in ADHD are a more plausible explanation. These impairments have found to be related to impairments in a range of developmental domains, such as social deficits (Sinzig et al., 2008) and motor coordination difficulties in ADHD (Fliers et al., 2008). Some studies suggest overlap between ASD, SLI and ADHD both at the phenotypic and the aetiological level, other studies however, indicate that different underlying mechanisms can lead to similar behavioral profiles. Examinations of similarities and differences in invariance detection between developmental disorders and the relationships of perceptual learning with development in different domains in these groups are needed to answer the question whether an invariance detection deficit is specific for ASD.

## Research Implications: Testing the Theory

The current theory predicts that individuals with ASD have specific difficulties with the perceptual learning process of detecting invariance over change compared to age and IQ matched controls. In contrast to other theories, our theory suggests a problem in a domain-general *learning process* instead of suggesting a static impairment. Tasks that are suitable to test our hypothesis are for instance category learning tasks that involves training individuals to classify abstract unfamiliar (novel) shapes or patterns of dots into categories (Dovgopoly and Mercado, 2013). It is best to use non-social tasks to exclude the explanation that the difficulties in performing this task in ASD can be attributed to social deficits, such as a lack of ToM (Baron-Cohen et al., 1985). Moreover, the invariant patterns in this task should consist of interchange between configural/global and local characteristics. We expect that individuals with ASD have difficulties in detecting the invariant patterns over change regardless of the nature (global or local) of the information. This excludes the possibility that the impairments can be attributed to deficit in WCC (Frith and Happé, 1994; Happé and Frith, 2006) or enhanced perceptual functioning (Mottron et al., 2006). In addition, by using unfamiliar novel shapes it is less plausible that the impairments in perceptual learning can be attributed to weak priors as suggested by Pellicano and Burr (2012). It is expected that performance on this task will be influence by length of exposure, length of training, and complexity in terms of the amount of variant properties. Individuals with ASD are expected to need more exposure and training in order to detect invariants, and they might be unable to do so in the most complex conditions. In addition, it is expected that performance on perceptual learning tasks is related to skills in different developmental domains. Longitudinal research is needed to examine whether individual differences in the ability to detect invariance may be related to the development of autistic behaviors, both talents and impairments, and whether these differences can attributed to differences in neural plasticity.

## Practical Implications

Since invariance detection is assumed to be a fundamental learning process that is already present at birth, a deficit in this learning process that can be detected with well-designed experimental research designs may provide opportunities for the early screening and detection of ASD. If our hypothesis is correct, than it is likely that individuals with ASD will benefit greatly if information is provided in an invariant, structured and predictable way. Robots might be able to provide such an environment (e.g., Srinivasan and Bhat, 2013). Parents and professionals may also address this need by adapting the physical environment and adjusting their behaviors and communication toward children and adults with ASD in a manner that limits variance. The proposed hypothesis of a deficit in invariance detection is in line with several studies that confirmed the utility of structuring as an intervention approach for children with ASD. The Treatment and Education of Autistic and Communication Handicapped Children (TEACCH; Mesibov et al., 2004) program emphasizes structure and has come to be called “structured teaching.” Important elements of structured teaching include organization of the physical environment, predictable sequence of activities, visual schedules and structured work/activity systems. Studies support the effectiveness of the TEACCH program (Ozonoff and Cathcart, 1998; Panerai et al., 2002). Since most individuals with ASD with normal or high intelligence are able to learn and use explicit strategies, while having difficulties with implicit learning, this strength can be used to help people with ASD in their daily functioning. This may, to a certain extent, compensate for deficits in invariance detection. For example, the interventions with social stories make implicit regularities in social interaction, explicit.

Possibilities for intervention at the neurobiological level have also been suggested. Since neural mechanisms of synchronization and connectivity may play a role in invariant detection, deep brain stimulation (DBS) or transcranial magnetic stimulation (TMS) might be applied in order to reorganize cortical activity. TMS uses low-powered magnetic fields to alter the firing of neurons in one part of the brain, and can be tuned to a specific brain region and to either increase or decrease activity. Studies have shown that low-frequency TMS minimized cortical responses to irrelevant stimuli and increased responses to relevant stimuli in ASD (Sokhadze et al., 2010) and that DBS might be helpful in treating disorders that are co-morbid with ASD, such as Obsessive-compulsive disorder (de Haan et al., 2013).

## Conclusion

In this article, we theorized that individuals with ASD are born with a deficit in invariance detection, which prevents them from perceiving and experiencing order in a constantly changing environment. In our view, the present work expands upon other ASD theories by suggesting a specific learning process from which to understand the emergence of ASD symptomatology. Our theory also adds to traditional theories by taking into account that cognition cannot be separated from perception and action and emerges in the person-environment system during interaction. The theory presented in this paper needs further quantification. Researchers should attempt to integrate neurobiological and behavioral research results. Much work is needed to specify the role of invariant detection for the developmental trajectories of individuals with ASD from birth onward.

### Conflict of Interest Statement

The authors declare that the research was conducted in the absence of any commercial or financial relationships that could be construed as a potential conflict of interest.

## Footnotes

1 http://www.nbcnews.com/id/35150832/ns/health-mental_health/#.VOpWUHyG91Z
